# High-Content *Aloe vera* Based Hydrogels: Physicochemical and Pharmaceutical Properties

**DOI:** 10.3390/polym15051312

**Published:** 2023-03-06

**Authors:** Mariana Chelu, Monica Popa, Emma Adriana Ozon, Jeanina Pandele Cusu, Mihai Anastasescu, Vasile Adrian Surdu, Jose Calderon Moreno, Adina Magdalena Musuc

**Affiliations:** 1 “Ilie Murgulescu” Institute of Physical Chemistry, 202 Spl. Independentei, 060021 Bucharest, Romania; 2 Department of Pharmaceutical Technology and Biopharmacy, Faculty of Pharmacy, Carol Davila University of Medicine and Pharmacy, 6 Traian Vuia Street, 020945 Bucharest, Romania; 3 Department of Science and Engineering of Oxide Materials and Nanomaterials, Faculty of Applied Chemistry and Materials Science University Politehnica of Bucharest, 060042 Bucharest, Romania

**Keywords:** hydrogel, *Aloe vera*, biomaterials, green synthesis, physicochemical characterization

## Abstract

The present research focuses on the physicochemical and pharmacotechnical properties of new hydrogels obtained using allantoin, xanthan gum, salicylic acid and different concentrations of *Aloe vera* (5, 10, 20% *w*/*v* in solution; 38, 56, 71 wt% in dry gels). The thermal behavior of *Aloe vera* composite hydrogels was studied using DSC and TG/DTG analyses. The chemical structure was investigated using different characterization methods (XRD, FTIR and Raman spectroscopies) and the morphology of the hydrogels was studied SEM and AFM microscopy. Pharmacotechnical evaluation on tensile strength and elongation, moisture content, swelling and spreadability was also completed. Physical evaluation confirmed that the appearance of the prepared *Aloe vera* based hydrogels was homogeneous and the color varied from pale beige to deep opaque beige with increasing *Aloe vera* concentration. All other evaluation parameters, e.g., pH, viscosity, spreadability and consistency were found to be adequate in all hydrogel formulations. SEM and AFM images show that the structure of the hydrogels condensed into homogeneous polymeric solids with the addition of *Aloe vera*, in accordance with the decrease in peak intensities observed via XRD analysis. These results suggest interactions between the hydrogel matrix and *Aloe vera* as observed via FTIR and TG/DTG and DSC analyses. Considering that *Aloe vera* content higher than 10% (*w*/*v*) did not stimulate further interactions, this formulation (FA-10) can be used for further biomedical applications.

## 1. Introduction

As modern wound care systems used for antibacterial applications, hydrogels are hydrophilic 3D network structures based on bioactive components that can contribute to effective wound healing and have good biocompatibility, adhesion, biodegradability and ease of use [1]. Due to their absorbent capacity, they can be used as promising materials for the healing and care of both superficial wounds containing a small amount of exudate and wounds with abundant exudate [2].

Currently, a performing wound care system should provide several benefits for both the patient and the clinical staff: (i) provide a protective anti-contamination, antibacterial and moisturizing environment in the wound area with good permeability and high-capacity absorption of exudate; (ii) be readily removed (or absorbed, as appropriate); and (iii) be refreshed without causing pain to patients [3,4,5]. In addition, these wound care systems should be easy to obtain and be biocompatible, fully biodegradable, and demonstrate excellent biomechanical and viscoelastic properties [6,7]. Bio-hydrogel materials are multifunctional systems that allow the release of their bio-active components for various therapeutic applications and are particularly useful in the treatment of long-healing wounds such as burns or bedsores [8].

Nowadays, green syntheses using plant extracts or phytochemicals have received appreciable attention due to the efficacy of their bioactivities, being seen as outstanding solutions to many of the limitations associated with physical and chemical synthesis methods. For therapeutic purposes and pharmacological applications, special attention has been directed to *Aloe vera* [9].

Historically, *Aloe vera* has been consistently useful in the treatment of wounds and burns due to its ability to stimulate healing, tissue regeneration and reduce scarring, noting that its pharmacological action includes strong anti-inflammatory and antibacterial activity [10,11]. Since ancient times, *Aloe vera* has been used for healing purposes by the Mesopotamian (its first mention), Egyptian and Greek civilizations [12]. The US Pharmacopoeia has officially recommended *Aloe vera* as an effective skin protector since the early 18th century [13]. *Aloe* leaf extract contains polysaccharides (e.g., acemannan, glucomannan) found in the inner parenchymal tissue of the leaf, as well as an orange latex-like exudate with a bitter taste due to the presence of aloin, aloe-emodin and related compounds. The *Aloe vera* gel inside the leaves contains about 96% water, the remaining 4% being several dozen different essential components [14]. *Aloe vera* gel has been shown to help increase the amount of collagen in wounds and promote wound healing [15,16,17].

The main biologically bioactive compounds contained in *Aloe vera* plants are polyphenols, carbohydrates (acetylated glucomannan, acetylated mannan, arabinogalactan, cellulose, galactogalacturan, pure mannan, xylan), phytosterols, glycoproteins, amino acids (alanine, arginine, aspartic acid, glutamic acid, glutamine, threonine, glycine, histidine, hydroxyproline, isoleucine, leucine, lysine, methionine, phenylalanine, proline, threonine, tyrosine, valine) and mineral elements (calcium, chlorine, chromium, copper, iron, magnesium, manganese, potassium, phosphorous, sodium and zinc) with good anti-inflammatory, analgesic and wound healing capabilities. In addition, the main components of *Aloe vera* have anti-diabetic, anti-carcinogenic, cardio-protective and digestive-protective and bone and skin-protective effects [18,19,20]. When applied to the skin, *Aloe vera* has traditionally been observed to reduce redness, swelling and pain caused by wounds and burns [20].

Allantoin is found in many different organisms, obtained as a result of cellular metabolism, through the transformation of uric acid through enzymatic or chemical oxidation reactions [21]. It is widely used as one of the bioactive ingredients that promote the healing of scar tissue and scars, in skin care products, and as a material for cosmetology and pharmaceutical products. The main therapeutic effects of allantoin are: helping to remove scar tissues and scars; protecting the skin from sunburn, cracks and fissures; restoring ordinary skin moisture and elasticity; and exhibiting antioxidant properties [22].

Xanthan gum is a high-molecular-weight extracellular microbial polysaccharide produced by the microorganism Xanthomonas campestris (a species of *Gram-negative* bacteria). For commercial purposes, it is manufactured via a fermentation process and has been widely used for biomedical applications in formulations containing pharmaceuticals, cosmetics and as an additive in various industrial products and food as well as food packaging, water-based paints, oil, oil recovery, and construction materials [23,24]. In tissue engineering applications, it has shown great potential and was therefore used here as a gelling and stabilizing agent for hydrogels.

Natural salicylic acid (β-hydroxy) is a bioactive, antimicrobial component that significantly contributes to cell regeneration and tissue damage repair. It is a standardized black willow extract with evidenced efficacy as a natural and gentler alternative to synthetic salicylic acid. In this study, it was used because of its antimicrobial action, which leads to healing, without the irritations usually associated with the synthetic version [25].

In the literature are many reports of numerous natural and synthetic polymer-based hydrogels used for a wide range of wounds [26,27]. Silva et al. developed 3D sponges using natural *Aloe vera* gel using a freeze-drying technique [28]. A thin layer of gellan gum was used to stabilize the *Aloe vera* matrix. The authors demonstrated the potential use of *Aloe vera* based sponges as promising candidates for biomedical applications. Singh et al. prepared a chitosan–*Aloe vera* nano-biocomposite dressing that demonstrated a strong acceleration of the wound closure rate. This was attributed to the presence of acemannan, the main component of *Aloe vera*. It also demonstrated anti-inflammatory characteristics and an immunomodulatory result by upregulating phagocytosis [29]. Koga et al. developed a new alginate gel based on *Aloe vera* crosslinked with zinc ions for wound dressing [30]. They demonstrated that the *Aloe* based alginate gel presented adequate malleability and mechanical strength, which made it as promising material for wound dressing. In addition, in vivo studies have been shown to increase the healing process of incisional skin wounds. Another study demonstrated the potential of chitosan-based hydrogels modified with *Aloe vera* juice obtained via UV radiation to support the wound healing process [31]. The introduction of *Aloe vera* juice into the hydrogel matrix increases its sorption properties and also the hydrogel shows a higher hydrophilicity.

The aim of this study was to combine the efficiency of natural plants as active metabolites and their bioavailability to obtain new hydrogels with different concentrations of *Aloe vera (Aloe Barbadensis)* as promising biotherapeutic agents and investigate their main physical, chemical and pharmacotechnical properties. The novelty of the present work is due to the preparation of new materials with higher content of *Aloe vera.* The combination of the four biomaterials (allantoin, xanthan gum, *Aloe vera* and salicylic acid) has never been studied in the literature. Subsequently, the hydrogels obtained based on *Aloe vera* were subjected to various analyses (FTIR, XRD, DSC, TG/DTG, SEM, Raman, AFM) aimed at establishing their physical and chemical properties, including pharmacotechnical evaluation for characteristics such as mechanical properties, pH, swelling ratio and spreadability.

## 2. Materials and Methods 

### 2.1. Materials

*Aloe Barbadensis* leaf powder, organic (commonly called *Aloe vera*) (100%), 200 times concentrated, obtained by freeze-drying *Aloe* leaves, certified as 100% organic by Ecocert Greenlife according to the COSMOS-standard, allantoin powder (min. 99.0%), xanthan gum, high-molecular weight, very pure form (91.0–108.0%), and aqueous extract of black willow bark (*Salix nigra* bark) (9.8–11.5%) were bought as raw materials from a commercial store of certified organic products (Elemental SRL, Oradea, Romania) and used as received. Deionized water was used throughout the study.

### 2.2. Synthesis of Hydrogels

In the preparation of three distinct hydrogel samples, 5% (*w*/*v*) allantoin was dispersed in deionized water at *T* = 80 °C in a water bath in each solution, with continuous stirring, until dissolved. Then, in the three solutions cooled to room temperature, different concentrations of lyophilized *Aloe vera* powder (5%, 10% and 20%) (*w*/*v*) were gradually added, followed by the addition of 1% (*w*/*v*) salicylic acid. The solutions were stirred for approximately 6 h for homogeneous mixing. Addition of 2% (*w*/*v*) xanthan gum resulted in rapid gelation of the three hydrogels. Finally, three homogeneous composite hydrogels containing different concentrations of *Aloe vera* were formed with a three-dimensional structure and identified as FA-5 (with 5% *w*/*v Aloe vera*); FA-10 (with 10% *w*/*v Aloe vera*) and FA-20 (with 20% *w*/*v Aloe vera*).

**Figure 1:**
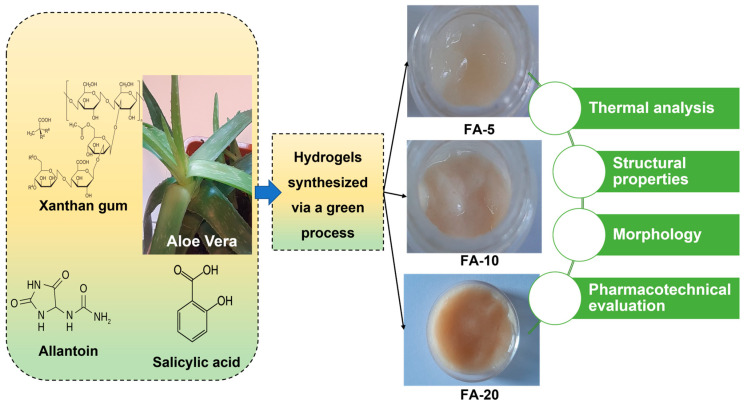
Scheme of synthesis, optical images of the three composite hydrogels with different *Aloe vera* concentrations (FA-5, FA-10 and FA-20) and their characterization.

Figure 1 shows the synthesis procedure of the composite hydrogel with the characterization methods.

### 2.3. Characterization Methods

#### 2.3.1. Visual Examination

The three composite hydrogel formulations prepared with different concentrations of *Aloe vera* were evaluated in terms of specific organoleptic properties such as appearance, consistency, color, homogeneity, and presence of any agglomerations or phase separation [32,33].

#### 2.3.2. Methods

Fourier transform infrared (FTIR) spectra were recorded using an Agilent 4100 ExoScan Spectrometer (Santa Clara, CA, USA) diffuse external reflectance system at the sample surfaces, between 400–4000 cm^−1^, with a resolution of 4 cm^−1^ and measurement time of 15 s.

X-ray diffraction spectra (XRD) were obtained using a PANalytical Empyrean diffractometer (Thermo Fisher, Waltham, MA, USA) equipped with a Cu X-ray tube (λ Cu Kα1 = 1.541874 Ǻ) at room temperature. The diffractometer was operated with in-line focusing, with a programmable divergent slit on the incident side and an anti-scatter slit mounted on the PIXcel3D detector on the diffracted side. XRD diffraction patterns were collected in a Bragg–Brentano geometry, using a scan step of 0.02° and a measuring time step of 2 s, from 20° to 80°.

Differential scanning calorimetry (DSC) analysis was recorded with a Mettler Toledo DSC 3 calorimeter (Mettler-Toledo GmbH, Greifensee, Switzerland), using a nitrogen gas flow of 80 mL min^−1^, in crimped Al pans with pierced lids. Thermogravimetric (TG) analysis and differential thermal gravimetry (DTG) were performed using a Mettler Toledo TGA/SDTA851^e^ instrument under a synthetic air flow rate of 80 mL min^−1^ using open alumina pans. Heating rates of 10 °C/min were used in both DSC and TG measurements.

Raman spectra were recorded in a Horiba Jobin Yvon LabRam HR spectrometer (Horiba, Ltd. Kyoto, Japan) using a 325 nm excitation laser and a NUV 40× objective.

For morphological analysis, the samples were left to dry and studied as such after drying. The morphology of the dried hydrogels was investigated using scanning electron microscopy (SEM) secondary electron images with a Quanta 3D field emission microscope (Thermo Fisher Scientific, GmbH, Dreieich, Germany) operating in high-vacuum mode at an accelerating voltage of 15 kV.

Atomic force microscopy (AFM) measurements were made using the XE-100 microscope from Park Systems (Park Systems Corporate, Suwon, Republic of Korea). All AFM scans were performed in non-contact mode with NCHR sharp tips from Nanosensors™ with a tip radius of less than 8 nm, ~125 μm length, ~30 μm width and ~42 N/m spring constant, and ~330 kHz resonance frequency. AFM images were recorded at two scales, namely (8 × 8) μm^2^ and (3 × 3) μm^2^. All images were processed with the image processing program XEI—v.1.8.0, developed by Park Systems for display purposes (1st order tilt correction) and roughness evaluation. Classical color AFM images (monochromatic brown gradient) as well as “enhanced color” (EC—“blue” images) are presented. Below the 2D EC AFM images, an arbitrary line (one-line scans collected along the X-scan direction) is presented for each surface, showing in detail the surface profile of each investigated surface.

#### 2.3.3. Pharmacotechnical Evaluation

##### Tensile Strength and Elongation Ability

The mechanical properties of the dry hydrogels were evaluated using a digital tensile force tester for universal materials (Lloyd Instruments Ltd., LR 10K Plus, West Sussex, UK). Measurements were recorded at a speed of 30 mm/min from a distance of 30 mm. The hydrogel was positioned vertically between the two plates and the breakage force was recorded. The tests were performed in triplicate and the mechanical attributes were calculated according to the following equations:

(1) $$\mathrm{Tensile}\;\mathrm{strength}\;\left(\mathrm{kg}/{\mathrm{mm}}^{2}\right)=\frac{\mathrm{Force}\;\mathrm{at}\;\mathrm{breakage}\;\left(\mathrm{kg}\right)}{\mathrm{Film}\;\mathrm{thickness}\;\left(\mathrm{mm}\right)\times \;\mathrm{Film}\;\mathrm{width}\;\left(\mathrm{mm}\right)}$$

(2) $$\mathrm{Elongation}\;\left(\%\right)=\frac{\mathrm{Increased}\;\mathrm{film}\;\mathrm{length}}{\mathrm{Inital}\;\mathrm{film}\;\mathrm{length}}\times 100$$

##### Moisture Content

Moisture content was determined via the thermogravimetric method [34] with a HR 73 Mettler Toledo halogen humidity analyzer from Mettler-Toledo GmbH, Greifensee, Switzerland. It was assessed on dry composite hydrogels and expressed as loss on drying (%).

##### pH Analysis

The pHs of the synthesized composite hydrogels were determined using a CONSORT P601 pH-meter (CONSORT^nv^, Turnhout, Belgium). We added 1 mL of distilled water (pH 6.5 ± 0.5) to the wet and dry hydrogel samples, and they were moistened at room temperature for 5 min [35]. Measurements are made for five experiments for each series, and pH values are expressed as mean ± SD.

##### Swelling Ratio 

Accurately weighed dry samples were fixed on 1.5% agar gel in Petri dishes and incubated at 37 ± 1 °C. For 6 h, every 30 min, the samples were weighed again. The swelling ratio was calculated according to Equation (3):

(3) $$\mathrm{Swelling}\;\mathrm{ratio}\;\left(\%\right)=\frac{W_{t}-W_{i}}{W_{i}}\times 100$$

where *W*_t_ is the sample weight at time t after incubation and *W*_i_ is the initial weight [36,37,38].

##### Spreadability

Spreadability of gels is an intrinsic attribute that links their rheological and structural characteristics. Because it can accurately predict behavior during product application and removal, it is a relevant test in evaluating semi-solid topical formulations. 

Spreadability analysis was performed on wet hydrogels using the extensiometric method that investigates their deformation ability when different weights are applied. Two square glass plates of 20 cm sides and 165 g were used. We placed 1 g of each sample on the lower plate, then the second glass plate was added and slightly pressed. After one minute, the diameter of the circle formed by the gel was measured. On the top plate of the extensiometer, weights of 50, 100, 200, and 500 g were progressively placed at one-minute intervals. Each time, the diameters of the circles created by sample spreading were registered [39,40]. Spreadability is expressed as the surface of the circles formed by the composite hydrogels, according to Equation (4):

(4) $$S=\pi r^{2}$$

where *S* is the spreading area (mm^2^) and *r* is the radius (mm).

## 3. Results and Discussion

The newly prepared composite hydrogels had a smooth texture and color ranging from pale translucent beige to deep opaque beige (Figure 1). Table 1 shows the organoleptic properties of the hydrogel composites with different content of *Aloe vera,* evaluated according to the specialized literature [32,33,41]. Optical and SEM studies demonstrated the uniform and consistent microstructure of the hydrogels. The hydrogels also exhibited consistent swelling, elasticity and mechanical strength behavior, shown below in Section 3.8 (at least three measurements were used for the mean and SD values).

### 3.1. FTIR Spectroscopy

**Figure 2:**
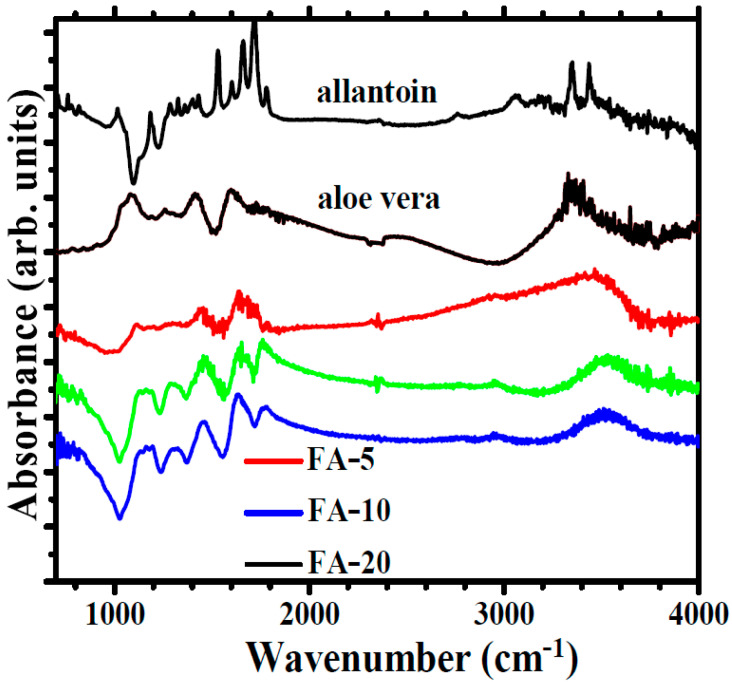
FTIR spectra of the raw materials (allantoin and *Aloe vera*) and the three formulations of *Aloe vera* based hydrogels (FA-5; FA-10; FA-20).

The FTIR spectra of the *Aloe vera* based hydrogels are presented in Figure 2.

The interpretation of the FTIR spectra was carried out based on the obtained results and by comparison with the data reported in the literature for different varieties of dry *Aloe vera* dried hydrogels. It should be noted that the FTIR analysis refers to the surface of the sample and allows the determination of the so-called short-range order at the molecular level.

The spectra of the three FA materials (Figure 2, FA-5, FA-10, FA-20) show similar features, with the vibrational bands reduced in intensity for the composition containing the least amount of *Aloe vera*. No significant differences were found between the FTIR spectra of FA-10 and FA-20 in spite of the increase in *Aloe vera* content. 

Six main functional groups centered at 3453, 1741, 1633, 1427, 1290 and 1170 cm^−1^ were identified in the spectra. There are also two weak bands observed at 2925 and 2755 cm^−1^. The FTIR spectra of *Aloe vera* precursor and allantoin are also shown for comparison in Figure 2. In the range of the 4000–2800 cm^−1^ spectral region, we observed essentially only the ν_OH_ and ν_CH2_ stretching vibrations. There is a broad band between 3600 and 3200 cm^−1^ centered at 3453 cm^−1^. This band is due to –OH stretching groups in the carbohydrate monomers from *Aloe vera* (e.g. uronic acid, mannonse or galacturonic acid, or phenolic constituents in antraquinones such as aloin, emodin), as in previously reported observations [44]. Such a wide range may also indicate the formation of intermolecular hydrogen bonds as a result of dipole–dipole attraction forces. It is still extremely difficult to find a reasonable correlation between these intense OH stretching bands and specific vibrational states. The broad OH stretching band is attributed, in agreement with data in the previous literature [44,45,46,47], to the contribution of various vibrational states of the complicated network of inter- and intramolecular hydrogen bonds occurring in the polysaccharide structure, especially the carbohydrate monomers of *Aloe vera* (uronic acid, galacturonic acid, phenolic compounds). Low-intensity bands in the range 2800–3000 cm^−1^ are associated with the asymmetric (2958 cm^−1^) and symmetric (2755 cm^−1^) C-H group stretching vibrations, respectively.

There are two band vibrations present in all spectra, centered at ~1760 cm^−1^ and at 1433 cm^−1^, characteristic of carbonyl stretching, corresponding to the asymmetrical and symmetrical -COO- of carboxylic acid (-COOH) groups, observed in other reported data too [48], due to the polysaccharides from *Aloe vera*. The high-intensity-mode band noticeable at about 1633 cm^−1^ corresponds to the C=O stretching of the carboxylic ester group—COOCH_3_, matching well with previously reported observations [48,49,50,51]. These functional groups are related to pectic substances, polysaccharides and water, the major compounds in *Aloe vera* based gels. Polysaccharides are the most abundant components in vegetal tissues. Generally, they can be divided into cell wall polysaccharides, such as cellulose, hemicelluloses and pectins, and storage polysaccharides such as acemannan, the main bioactive compound in the case of *Aloe vera* [52]. Commercial *Aloe vera* products are therefore desired to have a high polysaccharide content and this is also the main reason why the various effects of drying procedures—including preparation method, temperature, pH, O_2_ and the presence of other compounds—as well as the polysaccharides and their chemical and biological properties such as chain length, monosaccharide composition, emulsifying activity or structural conformation—are extensively studied [53]. In the range 1000–1300 cm^−1^, two intense and broad absorption bands are presented. Below 1300 cm^−1^, the -C(=O)-C stretching vibration modes of acetyl groups are observed. In the spectral region between 1000 and 1200 cm^−1^, the FTIR spectra of each polysaccharide fraction have a specific band maximum due to the C-O-C stretching vibration of the polysaccharide, which is dominated by ring vibrations overlapped with the (C-O-C) glycosidic bond vibration and the stretching modes of (C-O) side groups. This moiety, called the “saccharide band”, is sensitive to some conformational changes and is directly related to the crystal and amorphous phase in the samples [54]. Absorption bands characteristic to C-O-C stretching are associated with the multiple peaks in this band at around 1200, 1170 and 1128 cm^−1^ [10,55]. The bands at 1128 and 1170 cm^–1^ are attributed to C-O and C-OH bending vibration and determine the proportion of the crystalline phase [48]. The peak observed at ~1128 cm^−1^ can also be associated with the presence of C-O and C-OH bonds in both ester and ether overlapping modes from polysaccharides and alginates in *Aloe vera*. The bands at 789 and 840 cm^−1^ are attributed to out-of-plane C-H bending (mannose and or pyranose ring in *Aloe)* [56] and -CH_2_ twisting [57,58,59].

Similar absorption bands can be observed in all the compositions, with note that the vibrational bands in the samples with lower *Aloe vera* content are broader. Increasing the *Aloe vera* content made the broad bands clearer and better defined. From the FTIR analysis of the samples with different *Aloe vera* contents, we can conclude that amounts of *Aloe vera* up to 71 wt% in the composition of the dry gel FA-20 did not have a strong influence on the chemical structure of the gels. There are small variations in the intensity and location of the peaks, especially in the 900–1300 cm^−1^ region, which most likely results from the presence of many active substances.

### 3.2. XRD Analysis

XRD analysis was carried out to investigate the structure of *Aloe vera* based hydrogels. The XRD patterns of raw materials (allantoin, *Aloe vera*, xanthan gum) and the three composite *Aloe vera* based hydrogels are shown in Figure S1a,b in the Supplementary Materials File. The XRD spectrum of allantoin (Figure S1a in the Supplementary Materials File) exhibits intense diffraction peaks at 2θ = 15.9°, 20.5°, 22.2°, 24.7°, 28.2°, 29.8°, 32.17°, and 35.1°, indicating its crystalline structure, in agreement with data in the literature [60]. The XRD pattern of xanthan gum (Figure S1a in the Supplementary Materials File) and *Aloe vera* powder do not show sharp peaks, indicating their amorphous pattern. This agrees with the amorphous nature of xanthan gum and *Aloe vera* reported by other researchers [61,62,63,64,65].

### 3.3. Raman Analysis

**Figure 3:**
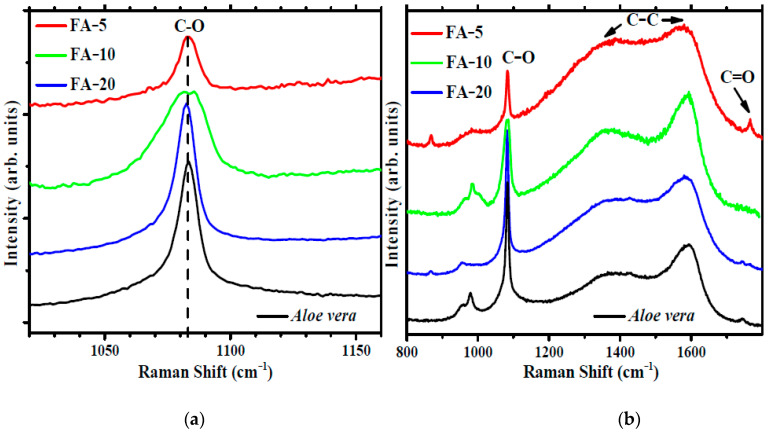
Raman spectra of FA-5 (red line), FA-10 (green line), FA-20 (blue line) and *Aloe vera* (**a**) and detail of the spectra in the region of the main characteristic band of acemannan (**b**).

Raman spectroscopy analysis of the dried gels was performed to identify the presence of different functional groups and types of bonding in each gel and compare their spectra. Figure 3 shows the Raman spectra of the FA materials and *Aloe vera* precursor powder.

The spectra are dominated by a strong feature at ~1083 ± 1 cm^−1^. This band corresponds to C-O stretching vibrations in the acemannan chain [46]. Acemannan is the main long-chain polysaccharide (carbohydrate) found in *Aloe vera* leaf gel [19] and one of the main bioactive components [20,66].

The relative intensity of the acemannan vibrational mode increases with *Aloe vera* content, while FA-5 and FA-20 display a sharp peak width at half peak ~10 cm^−1^, similar to that measured on the *Aloe vera* precursor. FA-10 presents a wider mode width at half peak ~25 cm^−1^. The contribution of other polysaccharides, such as galactose and mannose, mainly to the left of the peak at lower Raman shifts and the relative position of the side groups can influence the C-O stretching mode and cause the broadening of the acemannan mode [46]. Other strong Raman features are the D and G bands of C-C bonds between 1300 and 1700 cm^−1^ and a weak band observed at ~1766 cm^−1^, assigned to C=O stretching in acetylated polysaccharides. The intensity of this weak band appears to be associated with the intensity of another weak band at ~850 cm^−1^, attributed to the C-O-O stretching vibration of the ester groups, which can be used as indicative of the degree of esterification of the polysaccharide structures.

### 3.4. DSC Analysis

**Figure 4:**
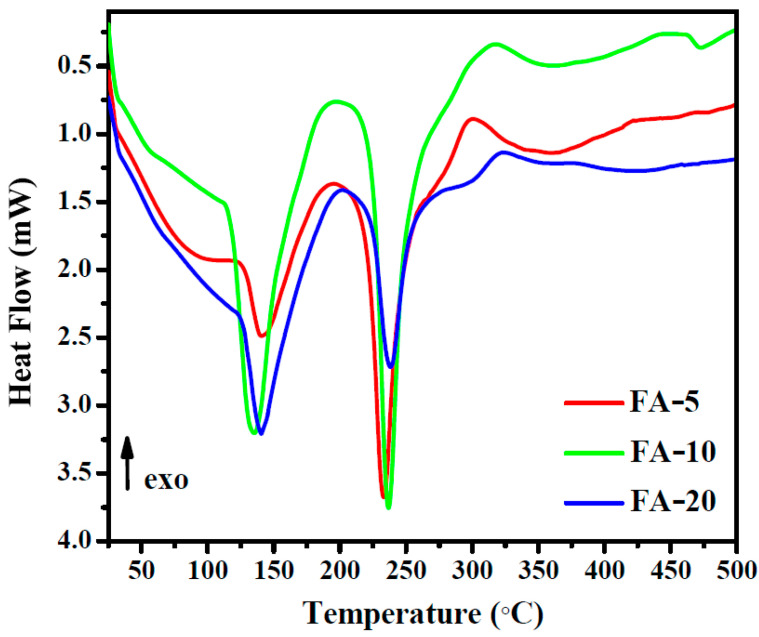
DSC curves of hydrogels containing 5, 10 and 20% (*w*/*v*) *Aloe vera*.

Figure 4 shows the DSC curves for the samples with different *Aloe vera* contents.

There is no distinct peak observed for the sublimation of salicylic acid, which occurs below 100 °C. The DSC curves show a strong endothermic peak around ~140 °C related to the vaporization of the volatile fraction, mainly water bound to the structure. This thermal event is also assessed in the TGA curves shown below in Section 3.5. A second endothermic effect in DSC is clearly observed for all samples at around ~230 °C and is associated with the thermal degradation of the polymeric composite. Yoshida et al. [67] observed two main endothermic peaks for *Aloe vera* extract at approximately 84.59 °C and 214.9 °C, while Garcia Orue [68] verified the presence of two endothermic peaks at 68 and 156 °C. In our research, we observed the endothermic effects of *Aloe vera* hydrogels at higher temperatures than in the studies mentioned above, with the appearance of an exothermic effect around 320 °C similar to the peak mentioned by Yoshida et al. [67]. This exothermic effect is slightly shifted to lower temperatures (320 °C) for the sample with lower *Aloe vera* content (FA-5).

### 3.5. Thermal analysis

**Figure 5:**
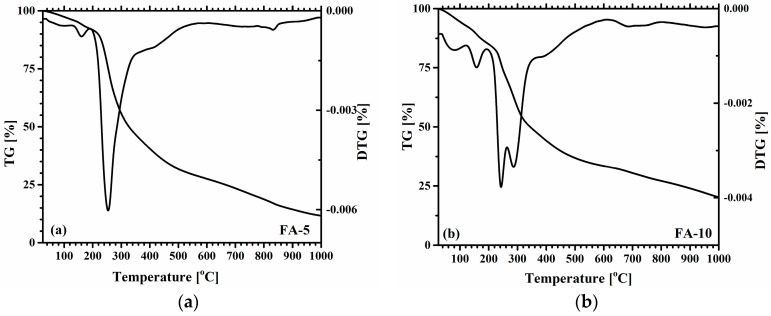

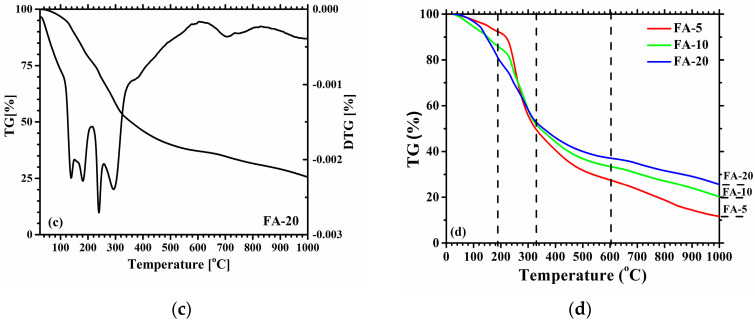
The TG/DTG curves for the three *Aloe vera* based hydrogels (**a**) FA-5; (**b**) FA-10; (**c**) FA-20. (**d**) Comparison of the TG behaviors.

Figure 5a–c shows the TG/DTG curves obtained for each of the three *Aloe vera* based hydrogels, while the TG curves are shown collectively for comparison in Figure 5d.

Thermogravimetric analysis was performed to evaluate the thermal stability of the samples, because together with DSC analysis, both methods help to understand the physical and chemical interaction of the compounds. 

It can be seen that the TG curves are similar and all hydrogels show multiple-stage thermal decomposition, with the main weight loss occurring between 200 and 300 °C. The main differences appear in the relative intensity of the thermal effects observed in the DTG curves accompanying the weight loss, highlighted in the sample with the highest *Aloe vera* content. The presence of a higher content of *Aloe vera* generated a slightly different thermal behavior in the gels, resulting in a wider range of thermal decomposition temperatures. As shown in Figure 5d, the TG/DTG curves present four different stages of thermal degradation, indicated in Figure 5d with dashed lines as a guide for the eye. There is a first stage that occurs in the temperature range 20–190 °C that corresponds to the evaporation of water and the volatile fraction. Two distinct bands can be distinguished in this region for the three DTG curves: a band peaking at 90–140 °C, with increasing intensity and temperature for increasing *Aloe vera* content, a clearly accentuated effect in FA-20 (Figure 5c); and a second peak, more intense, at 170 °C, also with increasing intensity with *Aloe vera* content. Gels belonging to polymeric materials contain different types of water, such as free water (usually released up to 60 °C), water interacting with hydroxyl groups, (usually lost up to 120 °C) and water bound to carboxyl groups (discharged up to 160 °C), which is consistent with our DSC results shown in Figure 4 and also in line with previous observations mentioned by Bialik et al. [45] and Avella et al. [69].

The second thermal stage, indicated by the dashed lines in Figure 5d, is the main thermal effect for all compositions. There is a visible drop in the TG curves of all three samples at ~240 °C, which can be related to the onset of thermal degradation of the polymeric composite. As the content of *Aloe vera* increases, the decomposition temperature shifts to higher temperatures and splits into two distinct peaks, an effect that can be attributed to the higher content of *Aloe vera*. While FA-5 shows a main thermal event centered at ~250 °C, FA-10 and FA-20 show a distinct separation of the main thermal effect into two separated peaks at 240 and 290 °C for FA-10 and FA-20.

The last two stages show thermal degradation steps starting at 400 °C, centered at 450 °C, and a final effect above 600 °C, indicating the occurrence of extensive degradation processes related to the complete decomposition of the polymeric structure into a carbon residue. The concentration of *Aloe vera* in the hydrogels can be associated with the increase in weight loss effects at temperatures lower than 200 °C and the different areas of thermal effects between the samples, due to the more salient contribution of the active substances polysaccharides and mucopolysaccharides present in *Aloe vera*; this effect can be observed at the intersection between the TG curves and the first dashed line in Figure 5d. The opposite trend can be observed between the concentration of *Aloe vera* and total weight loss. The presence of *Aloe vera* in the composition favors a reduction in total weight loss. The mass of solid residue after thermal analysis up to 1000 °C accounts for 11.61, 20.17 and 25.59% of the total mass of dry gels FA-5, FA-10 and FA-20, respectively. The solid residue mass and the *Aloe vera* concentration follow very closely a linear proportionality, this correlation clearly indicating that the residues correspond mainly to the carbon backbone remaining from the polymeric network of *Aloe vera* polysaccharides after thermal decomposition.

### 3.6. SEM Analysis

Morphological study via SEM revealed that all dried gels condensed into homogeneous polymeric solids (Figure S2 in the Supplementary Materials File). The structure of the gels was consistent at the microscale and the distribution of hydrogel components was uniform. All three materials have relatively dense surfaces. Sub-micron sized pores can be observed in the higher-magnification micrographs of FA-5 (inset in Figure S2c in the Supplementary Materials File).

### 3.7. AFM Results

**Figure 6:**
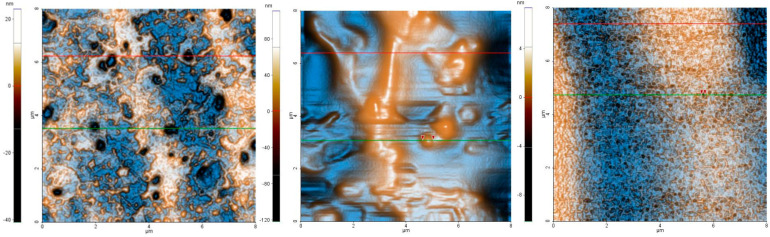

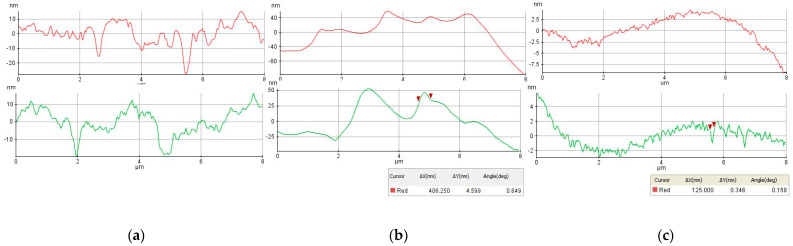
Enhanced color 2D AFM images at the scale of (8 µm × 8 µm), together with representative line scans for the aloe-based hydrogel samples: FA-5 (**a**), FA-10 (**b**) and FA-20 (**c**).

AFM was performed to determine the nanoscale surface topography. Figure 6 presents AFM images and line profiles of samples FA-5, FA-10 and FA-20 at the (8 µm × 8 µm) scale, as obtained from color-enhanced AFM images recorded in topographic mode. Based on the AFM images shown in Figure 6, it can be observed that the morphology of the *Aloe vera* based hydrogel samples is strongly dependent on their composition (*Aloe* content), as the FA-5 sample is smoother than the FA-10 sample and exhibits a structure of random shallow pores. These pores in sample FA-5 are several hundreds of nm in diameter, but an order of magnitude less in depth (tens of nm). Additionally, there is a distinctive texture consisting of small particles that are arranged individually or clustered together, creating the appearance of small, nanometer-sized fibrils (resulting in the corrugated shape of the cross-sectional height profiles of sample FA-5). In contrast, the FA-10 sample appears to be compact and shows some protruding crests (ridges) and a few particle-like features, such as the one marked along the green line, which is ~400 nm in diameter. Nevertheless, the scale of the cross-sectional height profiles differs in the two samples, as the z-scale of the two line-scans plotted for sample FA-5 are about 30–40 nm in height (~40 nm at the red line, from −30 to +10 nm, and ~30 nm at the green line, from −20 to +10 nm), while the z-scales of the two line-scans plotted for sample FA-10 are about ~100 nm high. 

For all areas scanned, the FA-5 sample has an RMS roughness of 6.5 nm and a peak-to-valley level difference of 63.8 nm, while the FA-10 sample has an RMS roughness of 36.1 nm and a peak-to-valley parameter of 232.7 nm. By further modification of the sample composition (increasing the *Aloe vera* content), the surface morphology is completely changed, leading to a smoother surface (with a lower roughness than even FA-5, which is the sample with the lowest *Aloe vera* content in this series) and exhibiting a “dense” structure of nanometer-sized surface pores. These pores, which are visible as small pits (dark spots) in Figure 6c, have diameters from tens to ~hundreds of nm (the largest), while their depth is a few nm. For example, the pore marked (by two red arrows) along the green line-scan in Figure 6c has a diameter of ~120 nm and a depth of ~3 nm. Over the entire scanned area in Figure 6c, which is (8 µm × 8 µm), the FA-20 sample has an RMS roughness of 2.1 nm and a peak-to-valley level difference of 17.5 nm.

**Figure 7:**
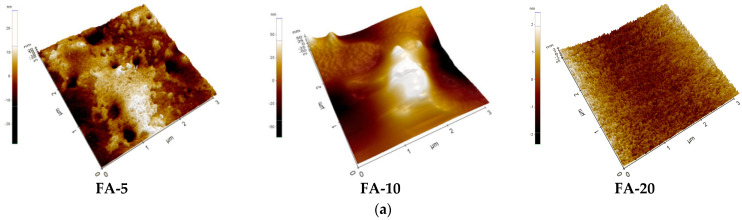

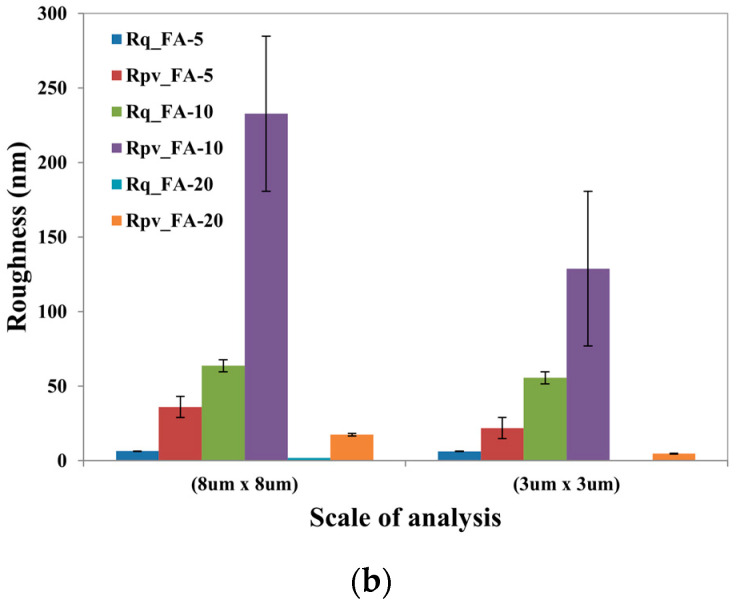
Three-dimensional AFM images at the scale of (3 µm × 3 µm) for the samples FA-5, FA-10 and FA-20 (**a**). Roughness histograms (RMS roughness―Rq and peak-to-valley―Rpv) for scanned samples at the two scales of analysis: (8 µm × 8 µm) and (3 µm × 3 µm) (**b**).

A detailed topographical view for samples FA-5 and FA-10 is presented in Figure 7a at the (3 µm × 3 µm) scale, based on the 3D AFM images. The corrugation behavior at both investigated scales is summarized in Figure 7b.

### 3.8. Pharmacotechnical Evaluation

#### Tensile Strength and Elongation Ability

The pharmacotechnical parameters are presented in Table 2.

**Table 2:** *Aloe vera* based hydrogel quality properties.

Tested Parameter *	Formulation Code					
	FA-5 Wet	FA-5 Dry	FA-10 Wet	FA-10 Dry	FA-20 Wet	FA-20 Dry
Tensile strength (kg/mm^2^)	-	1.06 ± 0.12	-	1.97 ± 0.23	-	1.08 ± 0.25
Elongation (%)	-	26.52 ± 0.34	-	47.83 ± 0.85	-	29.22 ± 0.76
Moisture content, % *(w/w)*	-	4.39 ± 0.84	-	7.27 ± 0.55	-	8.81± 0.35
pH	5.81 ± 0.03	5.85 ± 0.11	5.93 ± 0.01	5.96 ± 0.03	6.04 ± 0.06	6.05 ± 0.02
Swelling ratio (%, after 6 h)	-	187 ± 3.11	-	231 ± 4.79	-	212 ± 3.77

* Expressed as mean value ± SD.

Regarding the mechanical properties, a significant difference can be noticed in the behavior of the samples. This evidences the high influence of *Aloe vera* content on the hydrogels’ flexibility. FA-10 was found to have twice the strength and elasticity of FA-5 and FA-20, demonstrating that it will be easier to handle and apply. *Aloe vera* turned out to possess plasticizing properties in the gel structure, providing an enhancement in matrix density. FA-5 with an elongation of 26.52% and FA-20 with 29.22% were found to be less elastic than FA-10, whose elongation was found to be 47.83%. Bialik-Wąs K. et al. [45] found that increasing the concentration of *Aloe vera* leads to a decrease in tensile strength and elongation when using poly(vinyl alcohol) and sodium alginate as gel-forming polymers, associated with two plasticizers (poly(ethylene glycol) diacrylate and glycerin). Then, it must be recognized that *Aloe vera* interacts with the hydrogel matrix structure, producing different bonds and disruption in the polymer molecular chains depending on the type of polymer. It is clear that *Aloe vera* has an important contribution to the mechanical properties of the gels, and its performance depends not only on its concentration, but also on its interaction with the other components of the hydrogel. Still, the registered results demonstrate that the optimal *Aloe vera* concentration is 10 (*w*/*v*)% (56 wt% in dry hydrogels); meanwhile, a lower one, 5 (*w*/*v*)% (38 wt%), or a higher one, 20 (*w*/*v*)% (71 wt%), induce an obvious decrease in the cohesiveness of the dry hydrogels and the strength behavior.

The dry hydrogel FA-20 has the highest amount of moisture (8.81%) compared to the dry hydrogels FA-10 (7.27%) and FA-5 (4.39%). Considering the lack of a plasticizer in the formulation of the hydrogels, in spite of which FA-10 displayed acceptable elastic properties, it can be estimated that moisture content acts as a plasticizer in hydrogel structure. Londhe V. et al. [70] stated that dry hydrogels require a moderate quantity of moisture to maintain their elasticity and prevent them from becoming brittle and breakable. Still, in the case of FA-20, it can be noticed that a water content greater than 8% leads to a lower hardness and flexibility, attesting to the influence of the amount of *Aloe vera* on the pharmacotechnical properties of the hydrogels. These results confirm the report that the presence of moderate humidity is desirable. The increase in moisture content with increasing *Aloe vera* concentration for the studied hydrogels can be related to the dissolution of *Aloe vera* [71].

There were no differences between wet and dry hydrogels, nor between FA-5, FA-10 and FA-20, concerning pH values. This evidences that the pH value does not depend on *Aloe vera* concentration. All formulations presented slightly acidic characteristics compatible with the pH of the skin, indicating that the products will be well-tolerated and will not cause irritation.

**Figure 8:**
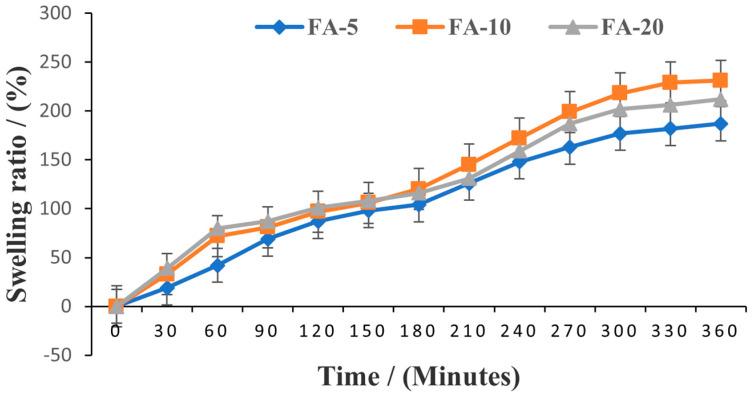
Swelling degree over 6 h of FA-5, FA-10 and FA-20 samples.

In view of the probable application of these composite hydrogels as wound care systems, swelling tests were conducted to determine the capacity of the hydrogels to absorb wound exudates. The degree of swelling over 6 h of the analyzed *Aloe vera* based hydrogel samples is shown in Figure 8.

Even though the matrix polymer was the same in all formulations, the absorption capacity turned out to be different between formulations. FA-5 containing a lower amount of *Aloe vera* displays lower swelling behavior (187%) compared to FA-10 (231%) and FA-20 (212%), demonstrating the clear influence of *Aloe vera* concentration on hydrogel expansion ability. It was shown that the swelling degree increased linearly in the first 5 h, after which the increase was slower, the differences between 300 and 360 min being almost insignificant. After 6 h, FA-5 and FA-20 eroded and the swelling ratio could not be determined. In contrast, FA-10 did not erode at the end of the study, but also no absorption could be detected after 6 h. These results indicate that the swelling reached its equilibrium level at 300 min and thereafter started to subside. These findings are in accordance with the conclusions reached by Bialik-Wąs K. et al. [45] that *Aloe vera* loosens the polymer network, allowing fluids to pass more easily between polymer chains and increasing swelling ability. Still, FA-20 turned out to be inferior to FA-10, showing that a higher concentration of *Aloe vera* does not necessarily lead to better performance and for a suitable formulation it is more important to select the most advantageous amounts of active ingredients. The results obtained suggest that the addition of *Aloe vera* at higher concentrations than 10% (*w*/*v*) did not affect the water uptake capacity of the hydrogels, a mandatory property for potential applications in wound care systems [65].

**Figure 9:**
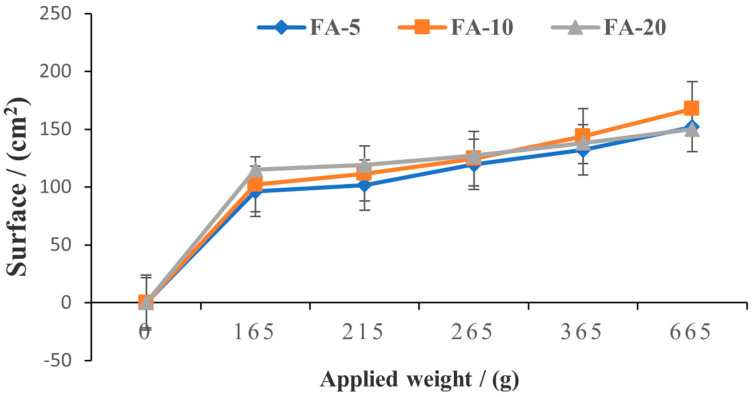
Spreadability of FA-5, FA-10 and FA-20 samples.

Figure 9 shows the variation in the surface occupied by 1 g of each *Aloe vera* based hydrogel depending on the applied weight.

Regarding spreading ability, the three hydrogels present similar behavior. Only a minor difference was detected, with an increased spreading area for FA-10, indicating that spreadability is primarily affected by the gel-forming polymer and only slightly by the amount of *Aloe vera* added. All formulations have adequate plasticity, making them spread across the skin with ease [72]. Typical hydrogel behavior was observed, with no significant differences when higher weights were applied. This evidences simple spreading on the skin when applying the studied hydrogels, without the need for massage opr force. The spreading characteristics demonstrate appropriate structural and viscoelastic properties for all three hydrogels.

## 4. Conclusions

Natural polymer-based hydrogels with high *Aloe vera* content were prepared in aqueous solution and as dry gels. The physicochemistries of the dry gels with different contents of *Aloe vera*, from 38% to 71% by weight, indicated the incorporation of allantoin and complex polysaccharides of *Aloe vera* in each gel. All dry gels were homogeneous, consistent composite polymers with smooth texture and color and no phase separation. The presence of *Aloe vera* conferred good consistency and plasticizing properties to the gel structure and also led to improved pharmacotechnical properties including swelling ratio, spreadibility, elasticity and tensile strength. The spreading characteristics demonstrate appropriate structural and viscoelastic properties for all hydrogels. The hydrogel with *Aloe vera* content of 10% (*w*/*v*) in solution and 55% by weight in dry gel exhibited the highest strength, elasticity and absorption capacity and also a slightly higher spreadibility, indicating it for application in wound care. The obtained results allow us to state that the newly prepared *Aloe vera* based hydrogels stand out for their high application potential as innovative wound care systems or transdermal systems that help the wound healing process due to the enhanced biomedical properties of *Aloe vera*.

## Figures and Tables

**Table 1:** Organoleptic evaluation of the prepared composite hydrogels.

Formulae	Appearances	Color	Homogeneity [42,43]	Consistency	*Aloe vera* Content		Phase Separation
					(*w*/*v*) %	Dried (wt%)	
FA-5	Homogeneous	Translucent, pale beige, neutral	Very good	Good	5	38	No phase separation
FA-10	Homogeneous	Opaque, natural beige	Very good	Good	10	55	No phase separation
FA-20	Homogeneous	Opaque, intense natural beige	Very good	Good	20	71	No phase separation

## Data Availability

Not applicable.

## Supplementary Materials

The following supporting information can be downloaded at: https://www.mdpi.com/article/10.3390/polym15051312/s1, Figure S1: XRD spectra of (a) raw materials (allantoin, *Aloe vera*, xanthan gum) and (b) *Aloe vera* based hydrogels (FA-5; FA-10; FA-20).; Figure S2: SEM images of (a,c) FA-5 and (b,d) FA-20 at different magnifications: (a,b) ×1000; (c,d) ×2000; and ×10,000 (inset in c).
