# Supplementary material for: High-Content Aloe vera Based Hydrogels: Physicochemical and Pharmaceutical Properties
Source: Polymers (Basel). 2023 Mar 6;15(5):1312. doi: 10.3390/polym15051312 (PMC10007233; doi:10.3390/polym15051312)
Supplement: Supplementary file 1 [file polymers-15-01312-s001.zip › polymers-2237404-supplementary.pdf]

Supplementary

High-Content *Aloe-vera*-Based Hydrogels: Physicochemical and Pharmaceutical Properties

Mariana Chelu [1,†], Monica Popa [1,*], Emma Adriana Ozon [2,†], Jeanina Pandele Cusu [1], Mihai Anastasescu [1], Vasile Adrian Surdu [3], Jose Calderon Moreno [1,*] and Adina Magdalena Musuc [1,*]

[1] "Ilie Murgulescu" Institute of Physical Chemistry, 202 Spl. Independentei, 060021 Bucharest, Romania
[2] Department of Pharmaceutical Technology and Biopharmacy, Faculty of Pharmacy, Carol Davila University of Medicine and Pharmacy, 6 Traian Vuia Street, 020945 Bucharest, Romania
[3] Department of Science and Engineering of Oxide Materials and Nanomaterials, Faculty of Applied
Chemistry and Materials Science University Politehnica of Bucharest, 060042 Bucharest, Romania
* Correspondence: pmonica@icf.ro (M.P.); calderon@icf.ro (J.C.M.); amusuc@icf.ro (A.M.M.)
† These authors contributed equally to this work.

Citation: Chelu, M.; Popa, M.; Ozon, E.A.; Cusu, J.P.; Anastasescu, M.; Surdu, V.A.; Calderon Moreno, J.; Musuc, A.M. High-Content *Aloe-vera*-Based Hydrogels: Physicochemical and Pharmaceutical Properties. *Polymers* **2023**, *15*, x. https://doi.org/10.3390/xxxxx

Academic Editor(s): Donatella Duraccio, Maria Giulia Faga, Giovanna Gomez D'Ayala

Received: 7 February 2023
Revised: 28 February 2023
Accepted: 3 March 2023
Published: date

Copyright: © 2023 by the authors. Submitted for possible open access publication under the terms and conditions of the Creative Commons Attribution (CC BY) license (https://creativecommons.org/licenses/by/4.0/).

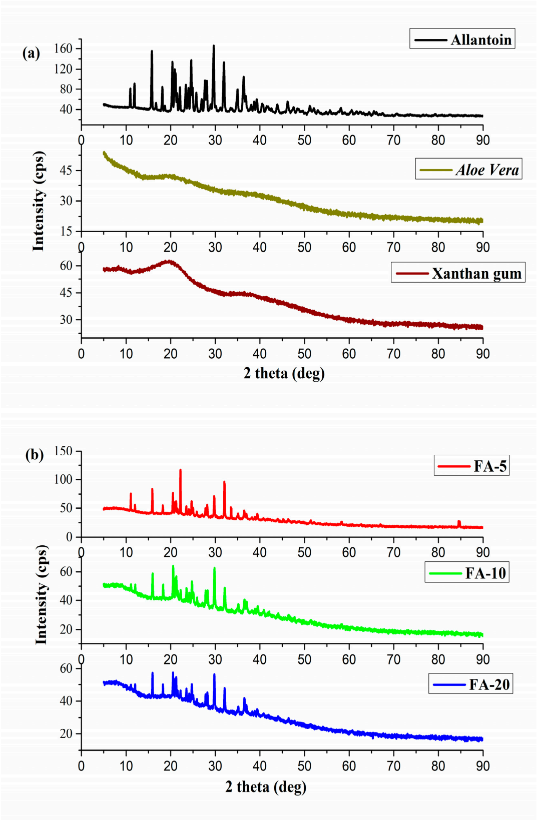

Figure S1. XRD spectra of (a) raw materials (allantoin, *Aloe vera*, xanthan gum) and (b) *Aloe vera* based hydrogels (FA-5; FA-10; FA-20).

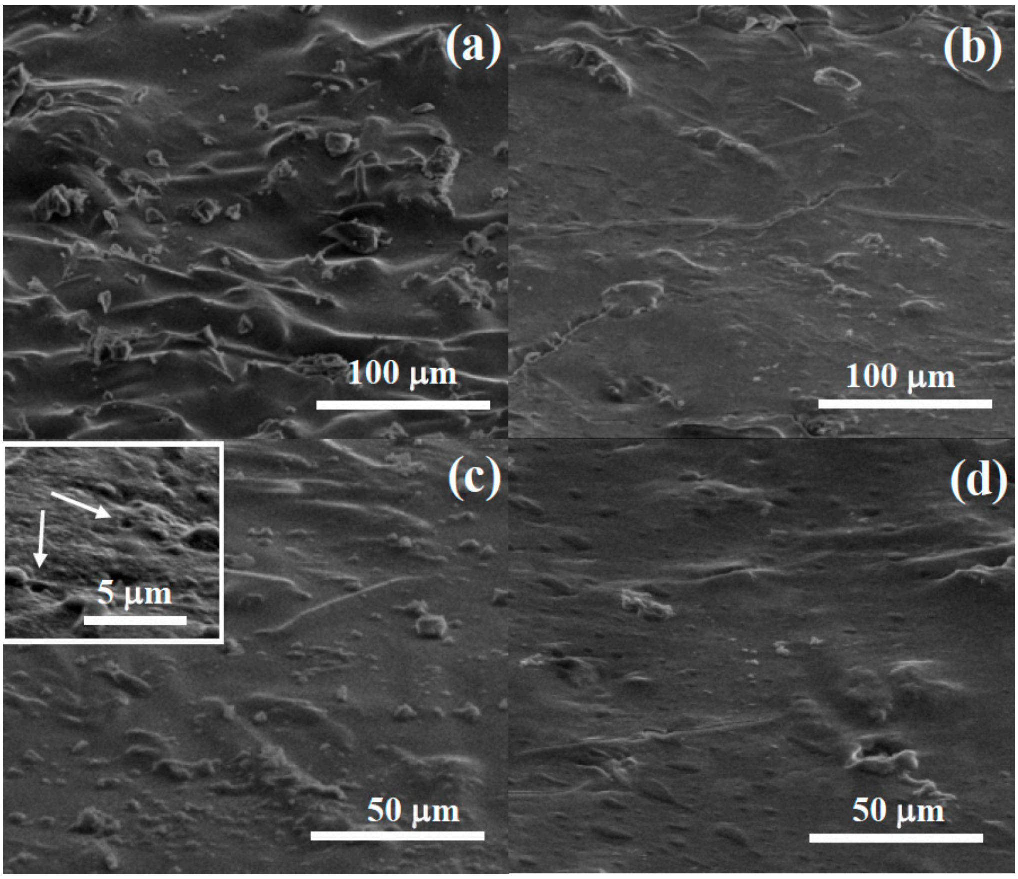

Figure S2. SEM images of (a, c) FA-5 and (b, d) FA-20 at different magnifications: (a-b) x1000; (c-d) x2000; and x10000 (inset in c).
